# Factors associated with nosocomial SARS-CoV transmission among healthcare workers in Hanoi, Vietnam, 2003

**DOI:** 10.1186/1471-2458-6-207

**Published:** 2006-08-14

**Authors:** Mary G Reynolds, Bach Huy Anh, Vu Hoang Thu, Joel M Montgomery, Daniel G Bausch, J Jina Shah, Susan Maloney, Katrin C Leitmeyer, Vu Quang Huy, Peter Horby, Aileen Y Plant, Timothy M Uyeki

**Affiliations:** 1 National Centers for Infectious Diseases, Centers for Disease Control and Prevention, Atlanta, GA, USA; 2 Department of Medicine, Hanoi Medical University, Hanoi, Vietnam; 3 Department of Medicine, French Hospital, Hanoi, Vietnam; 4 Department of Tropical Medicine, Tulane School of Public Health and Tropical Medicine, New Orleans, USA; 5 Centre for Biological Safety, Robert Koch Institut, Berlin, Germany; 6 Southeast Asia Regional Office, World Health Organization, Hanoi, Vietnam; 7 Division of Health Sciences, Curtin University of Technology, Australia

## Abstract

**Background:**

In March of 2003, an outbreak of Severe Acute Respiratory Syndrome (SARS) occurred in Northern Vietnam. This outbreak began when a traveler arriving from Hong Kong sought medical care at a small hospital (Hospital A) in Hanoi, initiating a serious and substantial transmission event within the hospital, and subsequent limited spread within the community.

**Methods:**

We surveyed Hospital A personnel for exposure to the index patient and for symptoms of disease during the outbreak. Additionally, serum specimens were collected and assayed for antibody to SARS-associated coronavirus (SARS-CoV) antibody and job-specific attack rates were calculated. A nested case-control analysis was performed to assess risk factors for acquiring SARS-CoV infection.

**Results:**

One hundred and fifty-three of 193 (79.3%) clinical and non-clinical staff consented to participate. Excluding job categories with <3 workers, the highest SARS attack rates occurred among nurses who worked in the outpatient and inpatient general wards (57.1, 47.4%, respectively). Nurses assigned to the operating room/intensive care unit, experienced the lowest attack rates (7.1%) among all clinical staff. Serologic evidence of SARS-CoV infection was detected in 4 individuals, including 2 non-clinical workers, who had not previously been identified as SARS cases; none reported having had fever or cough. Entering the index patient's room and having seen (viewed) the patient were the behaviors associated with highest risk for infection by univariate analysis (odds ratios 20.0, 14.0; 95% confidence intervals 4.1–97.1, 3.6–55.3, respectively).

**Conclusion:**

This study highlights job categories and activities associated with increased risk for SARS-CoV infection and demonstrates that a broad diversity of hospital workers may be vulnerable during an outbreak. These findings may help guide recommendations for the protection of vulnerable occupational groups and may have implications for other respiratory infections such as influenza.

## Background

**Figure 1:**
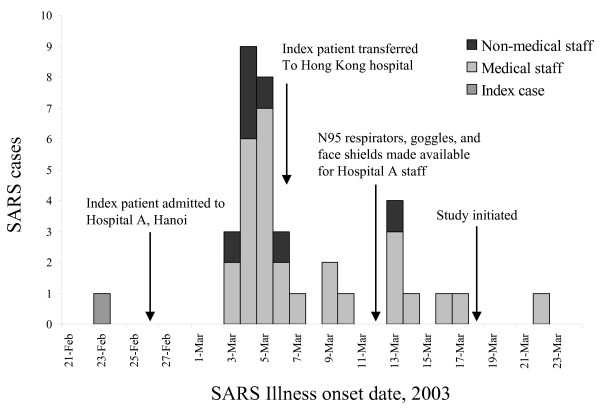
Epidemic curve of the SARS outbreak among Hospital A staff, Hanoi, 2003.

A World Health Organization (WHO) medical officer consulting with physicians at a hospital in Hanoi, Vietnam provided one of the earliest descriptions of a respiratory illness that later became known as SARS. Suspicions were first raised in Vietnam when staff at a small private health care facility, Hospital A, in Hanoi began to fall ill after caring for a business traveler recently arrived from Hong Kong [1]. This individual had been admitted to Hospital A on February 26^th ^(Figure 1), where he received care for 4 days in the general medical ward and 4 days in the intensive care unit. On March 5^th^, he was transferred to a facility in Hong Kong where he died one week later; he was subsequently confirmed to have SARS-associated coronavirus (SARS-CoV) infection. [2].

Enhanced infection control practices, cohorting of patients, and increased use of barrier protections were initiated on March 6^th^, after it was recognized that a severe respiratory illness was affecting several staff members. There were no negative pressure rooms at the hospital. N95 respirators, goggles, and face shields were made available to staff on March 12^th ^[*Uyeki, personal communication*]. Transmission of SARS-CoV among staff, visitors, and patients of Hospital A, and their close contacts outside the hospital, ultimately resulted in 62 cases of SARS in Northern Vietnam. Ninety percent of the 62 cases and all of the deaths (n = 6) occurred among individuals who visited or worked at Hospital A, which was temporarily closed on March 18^th^, 2003.

Hospital A is a small (<60 bed) private facility in Hanoi, providing inpatient specialist, laboratory, and nursing services. At the time of the outbreak, in addition to Vietnamese staff, several expatriate nurses and physicians were employed, often as clinical specialists on short-term contract from France. This report describes SARS-CoV secondary attack rates among the cohort of hospital staff at Hospital A and presents the results of a nested case-control study designed to identify risk-factors for SARS-CoV transmission after admission of the index patient. The primary objectives were to ascertain the extent of SARS-CoV transmission among the clinical and non-clinical staff at the hospital and to determine the nature of the initial exposures to the index patient that resulted in a substantial transmission event.

## Methods

### Cohort study population and enrollment

**Figure 2:**
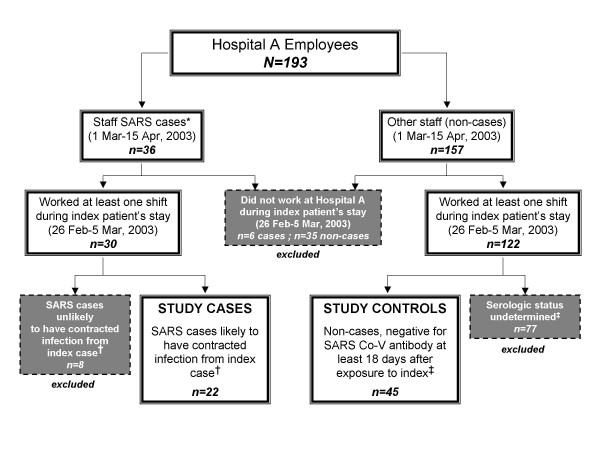
Diagram representing criteria for selection of case and control subjects to evaluate risks for SARS-CoV infection stemming from hospital exposure to the Hanoi index patient. (*) SARS cases were confirmed by serologic testing, viral culture, or RT-PCR performed on specimens obtained from persons with clinically compatible illness. (†) Excluded as study cases were SARS cases among the staff who were unlikely to have contracted infection from the index case (i.e., illness onset after March 5^th^, 2003 or seroconversion > 18 days after last exposure to the index patient); included as study cases are those SARS cases among the staff who had illness onset on or before Mar 5th, *or *seroconversion within 18 days of last exposure to the index case. (‡) Included as study controls were non-cases demonstrated to be negative for SARS-CoV antibody at least 18 days after last exposure to the index case. Potential control subjects were excluded from the analysis if no serologic specimen was collected from them or if the specimen was collected too early to assess final outcome status from exposure to the index case (i.e., within 18 days of last exposure).

We conducted a survey of symptoms and exposures among the cohort of workers at Hospital A, and a nested case-control study to assess risk factors for acquiring SARS-CoV infection after admission of the index case. For the latter study, only individuals who had worked at least one shift during the time the index patient was hospitalized were included (Figure 2).

During the outbreak, in the final two days that the hospital was open (March 17–18, 2003), investigators obtained a comprehensive list of hospital employees and held meetings with individual units (i.e., security, nursing, etc.) to recruit study participants. Upon providing written consent, participants were asked to complete a short, self-administered questionnaire and to provide a serum specimen for determination of antibodies to SARS-CoV. Questionnaires were translated into Vietnamese and responses were back-translated into English. Information was collected regarding the participant's contact with the index patient during his stay in the hospital from February 26^th ^(evening) to March 5th, as well as their symptoms from the time the index patient was admitted to the hospital until 10 days after he was transferred (~1.5 incubation periods [3-5]). Self-reported symptoms were checked against case investigation forms for 42 staff members. Participants were asked about whether they had ever engaged in a series of activities relating to exposure to the index patient. Questions were simplified in order to minimize translation errors, ensure that the activities would apply for multiple different staff job categories (e.g., nurse and security guard), and to facilitate self-administration.

All exposure variables were comprehensive for the period that the index patient was hospitalized at Hospital A. For example, *did you enter the general ward*, referred to whether or not the subject entered the general ward at least once at any time during the index patient's hospitalization. Variables were not all exclusive. For example subjects were asked whether they ever came within 1 meter of the index patient, and whether they ever did so without a mask (respirator). Further information pertaining to the number and duration of potential encounters was not requested due to time constraints and concerns about recall bias.

A physician who provided care for SARS patients at Hospital A throughout the outbreak (this physician resided temporarily at the hospital while the outbreak was ongoing) served as a proxy respondent for the staff who had died (n = 6) or were too ill (n = 1) to respond at the time of the survey. This physician had assisted WHO investigators in conducting the initial staff interviews in early March following recognition of respiratory illnesses among staff. Work shift schedules were available for 30.0% of the Hospital staff and were used to verify staff responses. The protocol was approved following expedited review by the hospital institutional review board and the Vietnamese Ministry of Health.

### Laboratory confirmation of cases

Laboratory confirmation of SARS-CoV infection was performed at the Centers for Disease Control and Prevention (Atlanta, Georgia, USA) and was based either on detection of RNA from SARS-CoV in clinical specimens (*via *reverse transcriptase polymerase chain reaction assays, RT-PCR), or by serology, as previously described [2]. Case definitions and laboratory assays employed during the outbreak in Vietnam are described in detail elsewhere [2].

### Nested case-control study

To determine which work-related activities may have been associated with contracting SARS-CoV infection after admission of the index case, a case-control study was performed. Inclusion and exclusion criteria for study cases and controls are outlined in Figure 2.

Study cases were defined as persons having: (*i*) the presence of SARS-CoV antibody in at least one serum specimen collected prior to March 24^th^; or (*ii*) for SARS cases confirmed by means other that serologic testing (i.e., viral culture or RT-PCR), illness onset on or before March 5^th^, the last day the index patient received care at Hospital A. SARS Co-V infections resulting from exposure to the index patient were presumed to have resulted in antibody conversion on or before March 24^th ^(18 days after the index patient's discharge) based on a conservative estimated timeline that includes a 6 day incubation period plus a 12 day interval to seroconversion. The estimated 6 day incubation period is based on the observed median number of days from exposure to illness onset among persons in Vietnam who reported only a single potential exposure to a SARS patient (n = 28). The 12 day estimated interval to seroconversion is based on the observed median number of days from illness onset to SARS-CoV antibody seroconversion among cases in Vietnam for whom the date of seroconversion is known within 48 hours (n = 17). This time frame is expected to exclude individuals who contracted infection from someone other than the index, while capturing the majority of individuals who were infected during index case's hospitalization in Hanoi, including a proportion of those who could have been infected on the last day of exposure to the index patient. The timing of illness onset alone was insufficient as a case selection criterion for the case-control study as this information was unavailable for many study enrollees.

Controls were defined as individuals within the study cohort who (*i*) were never identified as SARS cases, (*ii*) worked at least one shift during the time the index patient was hospitalized, and (*iii*) did not exhibit evidence of SARS-CoV specific antibodies within at least 18 days after last exposure to the index case.

### Statistical analyses

Exposure and demographic variables used for analyses were dichotomous categorical; unknown or missing data was rendered as a negative response. Non-parametric tests (*Fisher's exact*, *Cochran's Chi-square*) or odds ratios (OR) with 95% confidence intervals (CI) were used to assess differences between groups using negative responses as the referent. The threshold for statistical significance was established at a *p-value *< 0.05.

Advanced age (> 50 years) and underlying medical conditions were not significant correlates of case status in this outbreak [*Uyeki, personal communication*] and were therefore not included in the analysis. All statistical analyses were performed using SPSS version 11.0.1 (SPSS Inc., Chicago, IL).

## Results

### Survey of hospital workers

A staff census prepared in February 2003, indicated that 193 individuals were employed at Hospital A at the time of the SARS outbreak. The distribution of personnel between clinical and non-clinical roles is shown in Table 1. Twenty-nine of the 36 SARS cases (81%) at Hospital A occurred among clinical personnel with direct patient care or ancillary clinical roles. Direct patient care activities included primary medical functions such as patient examination and diagnostic evaluation, performance of procedures, and conduct of ongoing care and monitoring. In addition, non-clinical personnel were affected as well, with 19% of cases (n = 7) occurring among housekeepers and other cleaning staff (n = 5), kitchen staff (n = 1), and receptionists (n = 1). Several of the affected housekeepers entered the index patient's room on the night he was admitted to clean vomitus and respiratory secretions from the floor and walls, and various kitchen staff entered his room to deliver meals or collect service trays. Figure 1 depicts the epidemiologic curve of SARS illness among Hospital A personnel.

Two peaks of illness onset among staff were evident, on March 4^th^, and 9 days later on the 13^th ^(Figure 1). All but one of the non-clinical staff cases became ill during the first apparent wave of the outbreak, which encompassed the period the index patient was an inpatient at Hospital A. Housekeepers and certain members of the kitchen staff had access to the index patient's room(s) during his stay, for purposes of cleaning and meal delivery. Overall, observed attack rates were highest among personnel with direct patient care responsibilities. Notably, however, nursing staff at Hospital A who were assigned to the operating Room (Op.Rm.)/intensive care unit (ICU) had significantly lower attack rates (7.1, p < 0.006) than did nurses in other staffing categories. Though mid-wives might not be anticipated under normal circumstances to have had opportunities for direct contact with the index patient, several women who had had complicated deliveries were hospitalized in the inpatient ward or ICU during the index patient's stay; two briefly shared a room with him.

### Staff enrollment

Overall, 79% of Hospital A staff completed the exposure and symptom questionnaire, and 64% contributed at least one serum specimen. The lowest rates of participation in the exposure and symptom survey occurred among physicians and ICU nurses staffing categories (58.6%, 57.1%, respectively), but in both instances participation increased to over 64% for the serosurvey (Table 1). Staff work schedules were used, where possible, to independently verify a worker's presence at Hospital A during the index patient's hospitalization. A comparison between staff work schedules and worker responses is shown in Table 2. In general, there were fewer non-clinical staff than clinical staff participants in the serosurvey, but overall, serosurvey participation met or exceeded 50% in all but five staffing categories, these being, 'kitchen', 'dental', 'laboratory', 'administration', and 'reception'.

The questionnaire was completed by proxy for seven individuals who had died or were too ill to complete the questions at the time of survey administration. Removal of these respondents from the study pool did not substantively affect study findings (shown below), therefore these responses were included analyses as appropriate.

### Symptomatic illness among Hospital A staff

**Figure 3:**
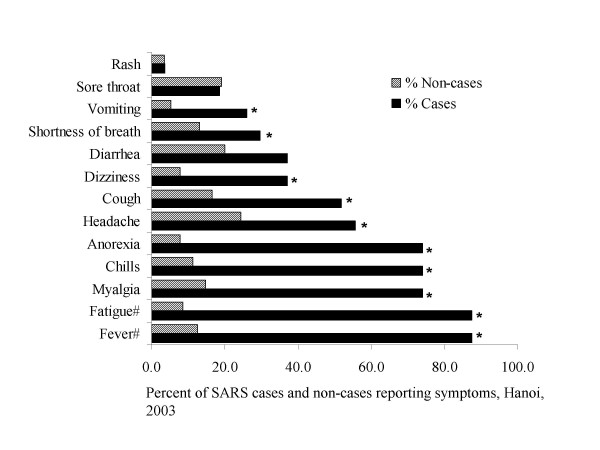
Subjective symptoms of illness reposted among staff at Hospital A (n = 27 SARS cases, n = 115 non-cases), Hanoi, 2003. The presence of an asterisk indicates that the symptom was significantly associated with SARS case status (p < 0.05, Fisher's exact, two-sided). (#) indicates sample sizes for cases and non-cases of 8, and 58 respectively.

Results of the survey regarding symptoms of illness experienced by study participants are summarized in Figure 3.

Consistent with findings from a clinical study of SARS patients conducted in Vietnam [6], fever, fatigue, myalgia, chills, anorexia, headache, and cough were the most frequently reported symptoms experienced by SARS infected staff at Hospital A. These symptoms occurred in over half of SARS cases, and all occurred at levels significantly above those reported by employees without SARS-CoV infection. Dizziness, shortness of breath and vomiting were also reported at significantly higher frequencies among SARS cases than uninfected staff, but nevertheless were reported by = 40% of SARS cases. Only diarrhea, sore throat, and rash were not reported at higher frequencies among SARS cases than uninfected staff. The background occurrence of rash was low among all participants, but both sore throat and diarrhea occurred at appreciable background rates (19% and 20%, respectively) among staff not infected with SARS-CoV.

### Serologic profiles of Hospital A employees

Of the 124 Hospital A staff who participated in the serosurvey, 36 (29%) had at least one serum specimen that tested positive for the presence of antibody to SARS-CoV antigen (Table 1). Four Hospital A staff members with confirmed SARS-CoV infection did not complete the questionnaire or participate in the serosurvey. All 32 confirmed SARS cases that did participate in the survey tested positive for the presence of antibody to SARS-CoV antigen. Four additional, previously unrecognized, seropositive individuals were identified as a consequence of the serosurvey. Thus, 4 of the 36 initially identified SARS cases during the outbreak did not have serological confirmation, and they were replaced by the 4 additional, previously unrecognized, seropositive individuals identified as a consequence of the serosurvey (n = 36).

These 4 newly identified seropositive individuals (2 general ward nurses, 1 laundry worker, and 1 receptionist) had mild illness and were not identified during the outbreak despite active surveillance conducted among staff at Hospital A. Among this group, the 2 General Ward nurses reported having had at least three symptoms associated with SARS illness in this outbreak (the first reported fatigue, headache, and shortness of breath; the second also reported headache along with myalgia, chills, dizziness, anorexia, and vomiting), but neither reported having experienced fever or cough, which were inclusion criteria in the WHO SARS case definition used at the time. Both nurses also reported experiencing diarrhea during the study period. However, diarrhea was common among all Hospital A workers (23% of all reporting workers) regardless of SARS case status. Neither the laundry worker nor the receptionist reported having experienced symptoms significantly associated with SARS illness in Vietnam, with the exception that the latter reported vomiting.

### Nested case-control study

Twenty-two study cases and 45 controls were identified (Figure 2). (Confirmed SARS cases not included in the study were excluded on the basis of illness onset date, date of seroconversion, or insufficient information to classify.)

Nearly all activities associated with physical proximity to the index patient or to his hospital rooms were significantly associated with SARS-CoV infection by univariate analysis (Table 3). However, touching the index patient (with or without personal protective equipment in the form of gloves, gown, face mask) or speaking to him in his room, were not significantly associated with SARS-CoV infection. Having had a job that involved direct patient care or sanitation/kitchen duties was also not associated with SARS-CoV infection. Having a non-clinical staff position was nominally protective (O.R. = 0.2, p = 0.011) against SARS-CoV infection, as was having had an upper-respiratory infection ('head cold', O.R. = 0.2, p = 0.039) within the prior 6 months.

Multivariate analyses were not performed due to the limited size of the study population.

## Discussion

SARS has been documented, under certain circumstances, to be highly communicable in hospital settings. Attack rates among workers with direct patient care roles have been observed as high as 10.0 and 11.8% in Canada and Hong Kong, respectively [5,7]. This study examined early events after the admission of a patient with SARS-CoV infection into a small hospital, at a time before the risks from unprotected exposure to SARS patients were fully appreciated. Our findings highlight the potential SARS transmission risks to hospital workers from potentially infectious surfaces and from proximity to a symptomatic patient.

During several other outbreaks, airborne transmission of SARS-CoV was suggested as an important route of transmission in hospitals and residential settings [8-10]. Our findings do not allow us to discriminate between potential transmission via large droplet versus dilute aerosols, although large scale transmission through a concentrated 'plume' of virus seems less likely here, as proximity to the index patient was nearly universal among those who were infected. These findings have important implications for worker protection, as many different categories of workers perform activities that may bring them into proximity with a SARS-CoV infected individual.

Overall, we found that hospital workers who had greatest opportunity for proximity to the index patient were clinical staff, and among clinical staff, those with direct patient care duties experienced the highest attack rates and death rates among all categories of Hospital A workers. When Hospital A closed during the SARS outbreak, a second hospital (Hospital B) was designated to care for suspect SARS cases. There were no SARS cases among staff at Hospital B, and a serosurvey conducted among workers at the facility revealed no inapparent or asymptomatic infections despite the presence of numerous confirmed SARS cases in the wards [1]. The reasons for the disparity in infection rates among staff between Hospitals A and B is unclear, but comparing outcomes between the two hospitals in general supports the importance of enhanced infection control measures, barrier protections, and patient isolation, as control measures within hospitals.

A previous study of clinical workers at Hospital A suggested that the proportion of doctors and nurses using masks as a precautionary measure increased significantly after the initiation of secondary cases and that the use of masks had a significant impact on diminishing SARS-CoV transmission [11]. The potential benefits of enhanced infection control practices and barrier protections are also in evidence when looking at differences among workers at Hospital A who provided direct patient care. Among nurses at Hospital A, those who administered clinical care to critically ill or post-operative patients (Op.Rm./ICU nurses) experienced the lowest attack rates among all nursing categories, and though few (3 of 8) reported having engaged in any of the activities identified as being associated with risk for SARS-CoV infection (Table 3), it is possible that the lower overall attack rate for this group of nurses reflects the fact that routine infection control precautions employed by Op.Rm./ICU to protect vulnerable patients in the ICU also provided these nurses with protection against exposure to SARS-CoV. Studies from other SARS outbreaks have shown that nurses charged with providing intensive care to patients experienced relatively fewer instances of SARS-CoV infection [5], unless assisting with a high risk procedure such as an endotrachael intubation [12,13].

During the course of this study, four workers, none of whom had been previously identified as cases, were found to have been infected with SARS-CoV. None of the four reported having had cough or fever, but two complained of diarrhea and another had a sore throat. A similar study conducted among health care workers in Singapore, revealed serologic evidence of SARS-CoV infection in two workers (of 112 exposed individuals) who experienced only mild symptoms of illness [14]. Both of these individuals had had fever and multiple systemic or upper respiratory symptoms, but neither developed pneumonia. Together, these reports and observations suggest that SARS-CoV infection can manifest with relatively mild symptoms, which can be easily masked against a background of unrelated illnesses in the community. The epidemiologic significance of mild (or asymptomatic) infections remains unclear however, and in this instance it is not known whether any of the four serologically positive individuals without pneumonia transmitted SARS-CoV to their contacts.

There were several limitations to this study. The first is the small sample size employed for the nested case-control, which contributed to a general lack of precision in measures of effect (odds ratios), and precluded our ability to look for independent risk factors through multivariate analyses. In addition, because of the need to minimize the complexity of the questionnaire, we were unable to assess either the duration, or the intensity of potential exposures, both of which are likely to be important modifiers of absolute risk. However, our streamlined approach using generalized questions allowed us to rapidly survey a large fraction of the hospital worker population, rather than just medical professionals. Finally, there were several potential sources of bias in this study which could have affected our results and conclusions. Although we performed the study prior to closure of the hospital, while the staff were still actively engaged in the outbreak investigation, we failed to achieve full staff participation, particularly among physicians and certain categories of nurses. This could have introduced a selection bias favoring enrollment of persons with less opportunity for direct contact with the index patient. Similarly, we questioned individuals about their exposure to the index patient 13 days after he was transferred to Hong Kong, and used a proxy to complete exposure questionnaires for deceased individual. Either of these could have introduced non-systematic information (recall) bias to our findings. We attempted to minimize the influence of these potential sources of bias by using case investigation forms and physician notes to verify self-reported information when ever possible.

Many of the job-related activities identified in this study as potential risk factors for SARS-CoV infection, such as entering the patient's room, and touching a visibly contaminated surface relate to 'proximity' contacts and possibly fomite involvement. These types of contact are broadly applicable to many different job categories from receptionist to physician, implying that our concept of occupational categories at risk for nosocomial infection may need to be broadened to include many different kinds of workers without direct patient care duties [5,7,15,16].

## Conclusion

The outbreak of SARS in northern Vietnam investigated here serves as a tragic reminder of the profound impact that the introduction of a highly communicable, virulent pathogen can have on the relatively closed community of a small hospital. In such instances, very early events following introduction can be pivotal in determining the ultimate magnitude of the outbreak and the degree of spread within the hospital. Appropriate recognition of those at highest risk of exposure and illness in conjunction with rapid, accurate identification of potential cases at the earliest stages of illness, are vital to minimizing the extent of spread. The results of this investigation highlight the diversity of workers at risk for nosocomial exposures and contribute to our understanding of risk factors for SARS-CoV transmission, which may include being in proximity to an infected patient or touching a contaminated surface.

## Competing interests

The author(s) declare that they have no competing interests.

## Authors' contributions

All authors read and approved the final manuscript. MR participated in the design of the study performed the statistical analyses and drafted the manuscript; BA participated in the design and coordinated implementation of the study; VT participated in the implementation of the study; JM participated in data analyses and preparation of the manuscript; DB participated in design of the study and preparation of the manuscript; JS participated in implementation of the study; SM participated in design of the study; KL participated in implementation of the study; VH participated in collection and analysis of laboratory diagnostic specimens; PH participated in data analysis and manuscript preparation; AP participated in design of the study; TU participated in design and implementation of study and supervised manuscript preparation.

## Pre-publication history

The pre-publication history for this paper can be accessed here:



## Figures and Tables

**Table 1:** Hospital A staff by job category – SARS attack rates and serologic profiles, Hanoi, 2003.

			**SARS outbreak at Hospital A**			**Enrollment number of hospital workers (%)**		
				
	**Staff position**	**Hospital A employees**	SARS cases*	**Attack rate^†^**	**Deaths**	**Completed survey**	**Serologic specimen**	**Enrolled workers with positive serologic result^‡^**
Direct patient care	Physician^§^	29	8	27.6	4	17 (58.6)	19 (65.5)	7 (36.8)
	Midwife	10	4	40.0	0	9 (90.0)	8 (80.0)	4 (50.0)
	Nurse^¶ ^(gen. ward)	19	9	47.4	2	16 (84.2)	19 (100)	10 (52.6) **2 new**
	Nurse^¶ ^(Op.Rm./ICU)	14	1	7.1	0	8 (57.1)	9 (64.3)	0
	Nurse^¶ ^(other)	7	4	57.1	0	7 (100)	6 (85.7)	4 (66.7)
*sub-total*		*79*	*26*	*32.9*	*6*	*58 (73.4)*	*61 *(77.2)	*26(42.6)*
Other clinical staff	Dental	3	0	0	0	3 (100)	1 (33.3)	0
	Laboratory	7	0	0	0	6 (85.7)	3 (42.9)	0
	Pharmacy	2	0	0	0	2 (100)	2 (100)	0
	Radiology	6	2	33.3	0	5 (83.3)	5 (83.3)	1 (20.0)
	Physiotherapy	1	1	100.0	0	1 (100)	1 (100)	1 (100)
*sub-total*		*19*	*3*	*15.8*	*0*	*15 (79.0)*	*12 *(63.2)	*2 (16.7)*
Sanitation/Kitchen	Housekeeping	16	4	25.0	0	16 (100)	11 (68.8)	4 (36.4)
	Laundry	7	0	0	0	7 (100)	6 (85.7)	1 (16.7) **1 new**
	Kitchen	5	1	20.0	0	4 (80.0)	2 (40.0)	1 (50.0)
	Other	2	1	50.0	0	1 (50)	1 (50.0)	1 (100)
*sub-total*		*30*	*6*	*20.0*	*0*	*27 (90.0)*	*20 (66.7)*	*6 (30.0)*
Other non-clinical	Administration	28	0	0	0	22 (78.6)	8 (28.6)	0
	Reception	13	1	9.1	0	10 (76.9)	6 (46.2)	2 (33.3) **1 new**
	Security	8	0	0	0	7 (87.5)	6 (75.0)	0
	Maintenance	3	0	0	0	3 (100)	3 (100)	0
	Operations	13	0	0	0	11 (84.6)	8 (61.5)	0
*sub-total*		*65*	*1*	*1.5*	*0*	*53 (81.5)*	*31 (47.7)*	*2 (6.5)*
**Total**	**Hospital A staff**	**193**	**36**	**18.7**	**6**	**153 (79.3)**	**124 (64.2)**	**36 (29.0)**

* Refers to cases identified during the course of the outbreak. 4 of the 36 initially identified SARS cases during the outbreak did not have serological confirmation, and they were replaced by the 4 additional, previously unrecognized, seropositive individuals identified during the serosurvey (n = 36).

† The case fatality rates among physicians, nurses from the general ward, and among staff with patient care duties are 50, 22, and 23%, respectively.

‡ Positive serologic results are listed as 'new' if the individual was not recognized as a symptomatic case during the outbreak, but was found to be seropositive during the course of this study.

§ Physicians cases include: Anesthetist/Anesthesiologist (3), General Practitioner (2), Pulmonary Specialist (1), Gynecologist (1), Orthopedic Surgeon (1). Radiologists are included in the 'Radiology' category.

¶ Nurses who were assigned to the general inpatient ward, or the intensive care unit/operating room are indicated, others not specifically assigned are grouped into a single category.

**Table 2:** Comparison of survey responses and staff work schedules during the SARS index patient's hospitalization in Hanoi, Vietnam.

		**Worked at least one shift during index patient's hospitalization, 26 February-5 March, 2003**		
			
**Staff position**	**Completed Survey n (%)**	**No. surveyed who reported working**	No. scheduled to work*	**Agreement (%)^†^**
Midwife (n = 10)	9 (90)	9	8	88.9
Nurse (Op.Rm./ICU) (n = 14)	7 (50)	6	6	100
Housekeeper (n = 16)	16 (100)	15	16	93.8
Receptionist (n = 10^‡^)	9 (90)	9	7	77.8
Security (n = 8)	7 (87.5)	7	7	100
**Total (n = 58)**	**48 (82.8)**	**46**	**44**	**95.7^§^**

* Shift schedules were obtained from Hospital A human resources administration for approximately 30% of Hospital A staff.

† Agreement between reported and scheduled work activity among surveyed staff.

‡ Receptionist does not include cashiers (1) and store clerks (2) who also worked in the reception area of Hospital A and who are included in Reception category in Table 1.

^§ ^Kappa score κ = 0.476 (p = 0.002); suggests intermediate qualitative agreement for two reporting sources.

**Table 3:** Single variable analysis of risk factors for SARS Co-V infection among hospital staff cases and controls, Hanoi, 2003.

				**SARS cases**		**Controls**	
				
**Activities During Index Patient's Hospitalization**	OR*	**95% CI**	***p-value^†^***	n = 22	%	n = 45	%
Touched index patient	2.8	0.9–8.5	0.085	9	(41)	9	(20)
Talked to or touched index patient without mask^‡ ^(ever)	1.9	0.6–5.9	0.363	7	(32)	9	(20)
Came within 1 meter of index patient	9.3	2.8–30.9	<0.001	17	(77)	12	(27)
Came within 1 meter of index patient, without mask^‡ ^(ever)	5.4	1.8–16.3	0.003	14	(64)	11	(24)
Spoke with index patient	3.5	1.2–10.4	0.028	11	(50)	10	(22)
Entered patient room	20.0	4.1–97.1	<0.001	20	(91)	15	(33)
Spoke with index patient in his room	3.7	1.1–12.6	0.052	8	(36)	6	(13)
Saw (viewed) index patient	14.0	3.6–55.3	<0.001	19	(86)	14	(31)
Visited patient room when patient was not there	3.7	1.3–10.9	0.027	12	(55)	11	(24)
Touched visibly contaminated surface	7.8	2.3–25.9	0.001	12	(55)	6	(13)
Entered general ward	8.0	1.7–38.4	0.005	20	(91)	25	(56)
**Other characteristics^§^**							
Upper respiratory infection w/in prior 6 months	0.2	0.04–0.9	0.039	2	(9)	15	(33)
'Other' non-clinical job^¶^	0.2	0.03–0.7	0.011	2	(9)	18	(40)
Direct patient care activities	2.0	0.7–5.6	0.298	13	(59)	19	(42)
Sanitation/kitchen job	2.2	0.7–7.0	0.223	7	(32)	8	(18)

* *Mantel-Haenszel *odds ratio; odds ratios refer to affirmative responses.

† *Fischer's exact *(two-sided).

‡ Respirator or surgical mask; N95 respirator masks were not widely available at Hospital A until March 12^th^.

§ A proportion of enrollees (n = 19 cases, n = 8 controls) were asked about a history of heart disease, lung disease, diabetes, and smoking. None of these factors had a statistically significant association with SARS cases status.

¶ Other non-clinical jobs include administration, reception, security, maintenance, operations (see Table 1).
